# Adipose-derived and bone marrow mesenchymal stem cells: a donor-matched comparison

**DOI:** 10.1186/s13287-018-0914-1

**Published:** 2018-06-19

**Authors:** Samih Mohamed-Ahmed, Inge Fristad, Stein Atle Lie, Salwa Suliman, Kamal Mustafa, Hallvard Vindenes, Shaza B. Idris

**Affiliations:** 1 Department of Clinical Dentistry, Faculty of Medicine, University of Bergen, Bergen, Norway; 2 Department for Plastic, Hand and Reconstructive Surgery, National Fire Damage Center, Bergen, Norway

**Keywords:** Adipose-derived stem cells, Bone marrow mesenchymal stem cells, Characterization, Proliferation, Differentiation

## Abstract

**Background:**

Adipose-derived stem cells (ASCs) have been introduced as an alternative to bone marrow mesenchymal stem cells (BMSCs) for cell-based therapy. However, different studies comparing ASCs and BMSCs have shown conflicting results. In fact, harvesting ASCs and BMSCs from different individuals might influence the results, making comparison difficult. Therefore, this study aimed to characterize donor-matched ASCs and BMSCs in order to investigate proliferation, differentiation potential and possible effects of donor variation on these mesenchymal stem cells (MSCs).

**Methods:**

Human bone marrow and adipose tissue samples were obtained from nine donors aged 8–14. ASCs and BMSCs were isolated and characterized based on expression of surface markers using flow cytometry. The proliferation up to 21 days was investigated. Multi-lineage differentiation was induced using osteogenic, chondrogenic and adipogenic differentiation media. Alkaline phosphatase (ALP) activity was monitored and collagen type I formation was evaluated by immunofluorescence staining. In vitro multi-potency was studied using tissue-specific stains and lineage-specific gene expression. In addition, the osteogenic lineage was evaluated at protein level.

**Results:**

Isolated ASCs and BMSCs from all donors demonstrated morphologic and immunophenotypic characteristics of MSCs, with expression of MSCs markers and negative expression of hematopoietic markers. Unlike BMSCs, ASCs showed high expression of CD49d and low expression of Stro-1. In general, ASCs showed significantly higher proliferation and adipogenic capacity with more lipid vesicle formation and expression of the adipogenesis-related genes than BMSCs. In contrast, BMSCs showed significantly higher osteogenic and chondrogenic capacity compared to ASCs. BMSCs had earlier and higher ALP activity, calcium deposition, and expression of the osteogenesis- and chondrogenesis-related genes and the osteogenesis-related protein osteopontin. Proliferation and differentiation capacity of ASCs and BMSCs varied significantly among the donors.

**Conclusions:**

ASCs and BMSCs showed tissue-specific differentiation abilities, but with significant variation between donors. The similarities and differences in the properties of ASCs and BMSCs should be taken into consideration when planning stem cell-based therapy.

## Background

Stem cell-based therapy has emerged as an alternative strategy in bone tissue engineering to overcome the limitations of autologous bone grafting. These limitations include donor site morbidity, risk of infection, nerve damage and hemorrhage [1]. Adult mesenchymal stem cells (MSCs) are undifferentiated multipotent cells characterized by the capacity for self-renewal and the ability to differentiate into various cells of mesenchymal origin, including adipocytes, chondrocytes, myocytes and osteoblasts, when exposed to specific growth signals [2]. MSCs can be obtained from different sources, including bone marrow, adipose tissue, dental pulp, synovium, muscle and other tissues [3]. The International Society for Cellular Therapy (ISCT) proposed that MSCs must be plastic-adherent (e.g. to a tissue culture flask), must express the surface markers CD73, CD90 and CD105 (≥ 90%), not express the hematopoietic markers CD14, CD34, CD45, CD19 and HLA-DR (≤ 2%), and should be able to undergo multi-lineage differentiation (osteogenic, adipogenic and chondrogenic) [4].

Bone marrow mesenchymal stem cells (BMSCs) have been the most extensively used and investigated MSCs. However, harvesting BMSCs has limitations due to the possible pain and morbidity associated with the bone marrow aspiration procedure and the limited number of MSCs obtained, as only a relatively small amount (0.001–0.01%) of the harvested bone marrow cells are MSCs [5]. BMSCs were also reported to show signs of senescence, early during expansion [6]. These issues have caused scientists to direct their efforts toward investigating alternative and comparable sources for MSCs. Abundant numbers of adipose-derived stem cells (ASCs) have been reported to be easily isolated from adipose tissue by a minimally invasive procedure [7]. In addition, ASCs can be obtained from adipose tissue from multiple sites [8–11]. Although significant biologic differences have been shown in MSCs derived from different sources [12–14], ASCs and BMSCs have comparable characteristics when it comes to morphology and surface proteins [15]. Further, ASCs have the ability to undergo multi-lineage differentiation, including osteogenic, chondrogenic, adipogenic, cardiomyocytic, hepatic and neurogenic differentiation [16]. The proliferation and differentiation capacity into different mesenchymal lineages make ASCs a promising less-invasive alternative to BMSCs for cell-based therapeutic applications [17, 18].

For bone tissue engineering, many studies have compared the in vitro osteogenic capacity of human BMSCs and ASCs [13, 18–25]. Some of these studies reported greater osteogenic capacity of BMSCs than ASCs [21–24], whereas other studies suggested that ASCs have equal or superior osteogenic potential compared to BMSCs [18–20, 25], making ASCs suitable for bone tissue engineering and osteogenic regenerative medicine [17, 26]. However, the majority of these studies compared ASCs and BMSCs obtained from different individuals. The proliferation and differentiation properties of MSCs obtained from one donor might differ from MSCs obtained from another donor [27], and age is considered to affect the properties of MSCs [28]. MSCs origin may be crucial for potential use in cell-based therapies. Studying donor-matched BMSCs and ASCs will result in a more reliable/robust comparison between these two types of MSCs. Therefore, the present study aimed to characterize donor-matched ASCs and BMSCs in order to investigate proliferation, differentiation potential and possible effects of donor variation on these MSCs.

## Methods

### Collection of adipose tissue and bone marrow aspirates

Human adipose tissue and bone marrow aspirates were obtained with informed parental consent from nine patients aged 8–14 years who had undergone iliac crest surgery for treatment of cleft lip and palate at the Department for Plastic, Hand and Reconstructive Surgery, National Fire Damage Center, Bergen, Norway.

### Isolation and expansion of human BMSCs

BMSCs were isolated from bone marrow aspirates and processed as previously described [29], with some modifications. In brief, 10 ml of human bone marrow was aspirated from the anterior iliac crest with the patient in a supine position. The aspiration syringe was loaded with 3000–5000 units heparin (Leo Pharma A/S, Ballerup, Denmark) to prevent clotting of the marrow sample. The aspirate was filtered with a 70 μm cell strainer, diluted 1:1 with culture medium [Dulbecco’s Modified Eagle’s medium (DMEM) (Invitrogen, Carlsbad, CA, USA) supplemented with 10% fetal bovine serum (FBS) (Hyclone - GE Healthcare Life Sciences, South Logan, UT, USA) and 1% antibiotics (penicillin/streptomycin; GE Healthcare Life Sciences)], and centrifuged at 1800 rpm for 10 min at room temperature (RT). After supernatant removal, the cell pellet was suspended in culture medium and plated in a 75 cm^2^ culture flask and maintained at 37 °C in a humidified atmosphere containing 5% CO_2_. After 24 h, cells were washed with phosphate-buffered saline (PBS) (Invitrogen) to remove the non-adherent cells. The culture medium was then changed twice a week. When 80% confluence was reached, cells were detached from the culture flasks using Trypsin/EDTA solution (Lonza, Basel, Switzerland). Cells were sub-cultured and expanded, using BMSCs at passage 3–5 for the in vitro assessment. Cell number and viability of BMSCs were assessed using 0.4% Trypan blue stain (Invitrogen) and a Countess™ Automated Cell Counter (Invitrogen). Growth and morphology of cells were routinely assessed using an inverted microscope (Nikon Eclipse TS100, Tokyo, Japan).

### Isolation and expansion of human ASCs

ASCs were isolated from subcutaneous adipose tissue as previously described [8]. In brief, adipose tissue block was extensively washed with PBS containing 5% antibiotics. The adipose tissue was minced and digested with 0.1% collagenase type I (Worthington Biochemical Corporation, Lakewood, NJ, USA) in PBS containing 2% antibiotics for 60 min at 37 °C. Collagenase was then neutralized with an equal amount of culture medium and centrifuged for 5 min at 2000 rpm. The centrifugation was repeated, preceded by shaking to disrupt the pellet, after which supernatant fluid was aspirated. The remaining pellet was suspended in culture medium, plated in a 75 cm^2^ culture flask and maintained at 37 °C in 5% CO_2_. Cells were expanded as described above, using ASCs at passage 3–5 for the in vitro assessment. Growth and morphology of cells were routinely assessed using the inverted microscope.

### Characterization of ASCs and BMSCs

ASCs and BMSCs characterization by flow cytometry was performed based on specific surface antigens. The cells were harvested and incubated with solutions of fluorescent antibodies, CD34, CD45, CD73, CD90, CD105, CD49d, HLA-DR (BD Biosciences. San Jose, CA, USA) and Stro-1 (Santa Cruz Biotechnology, Dallas, TX, USA) according to the manufacturer’s recommendations. Briefly, cells (4 × 10^5^) were suspended in 400 μl of PBS and centrifuged at 10,000 rpm for 5 min at 4 °C, and 20 μl of blocking reagent [0.5% bovine serum albumin (BSA) (Sigma-Aldrich, St Louis, MO, USA) in PBS] was added to the resulting cell pellet and incubated for 10 min at RT. Fluorescent monoclonal antibodies were added to each pellet and incubated in the dark for 30 min at 4 °C. Cells were then suspended in PBS, centrifuged at 10,000 rpm for 5 min and washed with PBS twice, followed by resuspension in 300 μl PBS. Final quantification was performed with a BD LSRFortessa cell analyzer (BD Biosciences). Stained samples (50,000 events) were analyzed and compared to the corresponding unstained samples. Data were analyzed using flow cytometry data analysis software (FlowJo V10, Flowjo, LLC, Ashland, OR, USA).

### Assessment of the metabolic activity of the cells

ASCs and BMSCs were cultured in 96-well plates (NUNC™, Thermo Fisher Scientific, Waltham, MA, USA) at a seeding density of 3 × 10^3^ cells/cm^2^ for 3, 7, 14 and 21 days. At each time point, cells were washed with PBS, and MTT solution [3-(4,5-dimethylthiazol-2-yl)-2,5-diphenyltetrazolium bromide solution (Sigma-Aldrich) in culture medium (dilution 1:4)] was added. The plates were incubated for 4 h in a humidified incubator at 37 °C containing 5% CO_2_. Cells were then fixed with Tris-buffered formalin for 5 min, washed with distilled water and left to dry overnight. Dimethyl sulfoxide (DMSO) (Sigma-Aldrich) was added and each plate was shaken on a plate shaker for 20 min before the absorbance was read at 570 nm using FLUOstar OPTIMA Microplate Reader (BMG Labtech, Offenburg, Germany).

### Multi-lineage differentiation of ASCs and BMSCs

For osteogenic differentiation, ASCs and BMSCs were seeded in 12-well plates at a density of 3 × 10^3^ cells/cm^2^. After 24 h, ASCs and BMSCs were washed with PBS and osteogenic differentiation medium was added. Osteogenic differentiation medium was prepared by adding 0.05 mM L-ascorbic acid 2-phosphate, 10 nM dexamethasone and 10 mM β glycerophosphate (all from Sigma-Aldrich) to the culture medium. ASCs and BMSCs in routine culture medium served as control. For chondrogenic differentiation, ASCs and BMSCs were seeded in 15 ml tubes at a density of 5 × 10^5^ cells to form a pellet. After 24 h, pellets were washed with PBS and StemPro® chondrogenic differentiation medium (Invitrogen) was added. BMSCs and ASCs pellets in routine culture medium served as control. All media were changed twice per week for 4 weeks. For adipogenic differentiation, ASCs and BMSCs were seeded in 12-well plates at a density of 7 × 10^3^ cells/cm^2^. After 24 h, ASCs and BMSCs were washed with PBS and StemPro® adipogenic differentiation medium (Invitrogen) was added. ASCs and BMSCs in routine culture medium served as control. Adipogenic and control media were changed twice per week for 2 weeks.

### Alkaline phosphatase (ALP) staining and assay

ASCs and BMSCs in osteogenic and control media were fixed with paraformaldehyde 4% at day 3, 7 and 14 for ALP staining, using SIGMAFASTTM BCIP/NBT tablets (Sigma-Aldrich). Images of the staining were taken using the inverted microscope. For the ALP assay at day 14, ASCs and BMSCs were lysed in 0.1% Triton-X100 buffer (Sigma-Aldrich), followed by two freezing-thawing cycles at − 80 °C, after which 20 μl of cell lysate was added in 96-well plate and mixed with 90 μl of working solution containing Sigma 104^®^ phosphatase substrate (Sigma-Aldrich) and alkaline buffer solution (Sigma-Aldrich). After incubation at 37 °C for 15 min, 50 μl of NaOH (sodium hydroxide) was added to stop the reaction. Absorbance was measured at 405 nm using the microplate reader. ALP activity assay was presented relative to BMSCs cultured in control medium as control samples.

### Immunofluorescence (IF) staining

Expression of collagen type I in ASCs and BMSCs after 14 days in osteogenic and control media was studied by IF staining. Cells were fixed with 4% paraformaldehyde for 15 min at RT, permeabilized with 0.1% Triton X-100 and blocked with 1% BSA in PBS. Cells were incubated under shaking with rabbit polyclonal anti-collage type I (Abcam, Cambridge, UK, dilution 1:500) overnight at 4 °C. Goat anti-rabbit Alexa Fluor 546 IgG was used as secondary antibody (Life Technologies, Carlsbad, CA, USA, dilution 1:800). The actin cytoskeleton was simultaneously stained for 45 min using phalloidin-Atto488 (Sigma-Aldrich, dilution 1:50). After washing with PBS, the nuclei were stained with 4′,6-diamidino-2-phenylindole (DAPI) (Sigma-Aldrich, dilution 1:2000). Images were taken using an inverted fluorescent microscope (Nikon Eclipse Ti, Tokyo, Japan).

### Evaluation of proliferation and multi-potency at gene level

Cells in osteogenic (day 7, 14 and 21), chondrogenic (day 28), adipogenic (day 14) and control media were harvested for RNA extraction. Total RNA was extracted using RNA extraction kit (Maxwell®, Promega, Madison, WI, USA) following the manufacturer’s protocol. Quantity and purity of RNA were determined by Nanodrop ND-1000 Spectrophotometer (Nanodrop Technologies, Wilmington, DE, USA). cDNA synthesis was achieved from 400 ng of total RNA using a High-Capacity cDNA Reverse Transcription Kit (Applied Biosystems, Foster City, CA, USA) according to the manufacturer’s protocol. Real-time quantitative polymerase chain reaction (qPCR) was performed using TaqMan Fast Universal PCR Master Mix (Applied Biosystems) following the manufacturer’s protocol. Amplification was performed in a 96-well thermal cycle plate on StepOne™ Real-Time PCR System (Applied Biosystems) to detect the gene expression of the proliferating cell nuclear antigen (PCNA). The osteogenic genes runt-related transcription factor 2 (Runx2), collagen type I, ALP and osteopontin, the chondrogenic gene aggrecan and the adipogenic genes peroxisome proliferator activated receptor gamma (PPARG) and lipoprotein lipase (LPL) were used to evaluate the osteogenic, chondrogenic and adipogenic differentiation. Glyceraldehyde-3-phosphate dehydrogenase (GAPDH) was used as an endogenous control. All gene primers were from Applied Biosystems (Table 1). Thermo-cycling conditions were 95 °C for 20 s, followed by 40 cycles at 95 °C for 1 s and 60 °C for 20 s. The expression of PCNA was presented relative to BMSCs in routine culture medium at day 7. The expression of the osteogenic genes Runx2, collagen type I, ALP and osteopontin was presented relative to BMSCs in osteogenic medium at day 7. The expression of the chondrogenic gene aggrecan and the adipogenic genes LPL and PPARG was presented relative to BMSCs in chondrogenic and adipogenic media, respectively. Data were analyzed by the 2^-Δ∆CT^ method.

**Table 1:** Real-time qPCR primers

Gene	Assay ID	Amplicon length
GAPDH	GAPDH: Hs 02758991_g1	93
PCNA	PCNA: Hs99999177_g1	69
Runx2	RUNX2: Hs01047973_m1	86
Collagen type 1	COL1A2: Hs00164099_m1	68
ALP	ALPL: Hs01029144_m1	79
Osteopontin	SPP1: Hs00959010_m1	84
Aggrecan	ACAN: Hs00153936_m1	91
PPARG	PPARG: Hs00234592_m1	77
LPL	LPL: Hs00173425_m1	103

*ALP* alkaline phosphatase, *GAPDH* glyceraldehyde-3-phosphate dehydrogenase, *LPL* lipoprotein lipase, *PCNA* proliferating cell nuclear antigen, *PPARG p*eroxisome proliferator activated receptor gamma, *qPCR* quantitative polymerase chain reaction, *Runx2 r*unt-related transcription factor 2

### Western blot analysis

For western blot analysis at day 21 in osteogenic medium, ASCs and BMSCs were lysed in a ready-made 2× laemmli sample buffer (Bio-Rad Laboratories, Hercules, CA, USA) supplemented with 2-mercaptoethanol (Sigma-Aldrich). Homogenates were collected, heated at 95 °C for 5 min and centrifuged at 16,000 rpm for 1 min. Each sample (15 μl) was loaded into 10% casted Sodium Dodecyl Sulfate (SDS)-Polyacrylamide Gel for electrophoresis, and then blotted to a Polyvinylidene Difluoride (PVDF) membrane (Bio-Rad Laboratories). The membrane was blocked with 5% skim milk (Sigma-Aldrich) for 1 h at RT, incubated with osteopontin primary antibody (sc-21,742, Santa Cruz, dilution 1:200) and β-actin antibody (sc-47,778, Santa Cruz, dilution 1:200) for 2 h at RT, followed by incubation with horseradish peroxidase-conjugated anti IgG secondary antibody (sc-516,102, Santa Cruz, dilution 1:1000) for 1 h at RT. Protein bands were visualized with Clarity Max™ Western ECL Blotting Substrate (Bio-Rad Laboratories) using ChemiDoc™ XRS+ system (Bio-Rad Laboratories).

### Evaluation of multi-potency using tissue-specific staining

For osteogenic differentiation, ASCs and BMSCs in osteogenic and control media were fixed with 4% paraformaldehyde at day 21 and 28 to evaluate calcium deposition using Alizarin red S staining. Briefly, after fixation, cells were stained with 2% Alizarin red S (Sigma-Aldrich) solution for 30 min at RT, and then washed and dried overnight. Images were made using the inverted microscope. For quantification, the stain was dissolved in cetylpyridinium chloride (Sigma-Aldrich) and absorbance was measured using the microplate reader at 540 nm. For chondrogenic differentiation, ASC and BMSC pellets in chondrogenic and control media were fixed with 4% paraformaldehyde at day 28. Alcian blue staining was used to examine the cartilaginous proteoglycan matrix formation. Briefly, cell pellets were embedded in paraffin and sectioned in 6 μm sections. Sections were stained with 2% Alcian blue stain (Sigma-Aldrich) in 3% acetic acid solution (Sigma-Aldrich) for 30 min and a 0.1% nuclear fast red (Sigma-Aldrich) solution was used as a counterstain. Images were taken using an upright microscope (Nikon Eclipse 80i, Tokyo, Japan). For adipogenic differentiation, ASCs and BMSCs in adipogenic and control media were fixed with 4% paraformaldehyde at day 14 to assess intracellular lipid vesicles using Oil red O staining. Briefly, 60% isopropanol (Sigma-Aldrich) was added to the wells and incubated for 5 min at RT. The isopropanol was removed and cells were stained with 0.3% Oil red O (Sigma-Aldrich) for 15 min. After washing, hematoxylin was added for 1 min to counterstain the cells before imaging, using the inverted microscope. The stain was then extracted using 99% isopropanol (Sigma-Aldrich) and quantified using the microplate reader at 540 nm absorbance.

### Statistics

Mixed-effects models for continuous data were applied for the statistical analyses. Since donor-matched ASCs and BMSCs were obtained from nine donors, donor was included in the models as a random effect. The results from the mixed models are shown in the figures as mean values with standard errors. Each donor is represented in the figures by a symbol (Table 2). *P* values less than 0.05 were considered statistically significant and are indicated by an asterisk in figures and tables illustrating the results. Intra-class correlations (ICC), based on the mixed models, were calculated to estimate the effect of donor on both types of cells. Data were analyzed using STATA (version 15, StataCorp, College Station, TX, USA).

**Table 2:** Comparison of the proliferation, osteogenic and adipogenic capacity of ASCs and BMSCs within each donor

Donor	Donor symbol	Age (years)	Gender	Proliferation 21 days	Osteogenic capacity			Adipogenic capacity
					ALP activity 14 days	Calcium deposition		Lipid vesicles formation 14 days
						21 days	28 days	
1		9	M	ASCs < BMSCs	ASCs > BMSCs	ASCs < BMSCs***	ASCs < BMSCs***	ASCs > BMSCs**
2		10	M	ASCs > BMSCs	ASCs < BMSCs	ASCs < BMSCs***	ASCs < BMSCs***	ASCs > BMSCs***
3		8	F	ASCs > BMSCs***	ASCs > BMSCs	ASCs < BMSCs***	ASCs > BMSCs	ASCs > BMSCs***
4		10	M	ASCs > BMSCs***	ASCs > BMSCs**	ASCs > BMSCs***	ASCs > BMSCs***	ASCs > BMSCs***
5		12	M	ASCs > BMSCs***	ASCs > BMSCs*	ASCs < BMSCs***	ASCs < BMSCs	ASCs > BMSCs***
6		11	M	ASCs > BMSCs	ASCs > BMSCs***	ASCs > BMSCs	ASCs > BMSCs***	ASCs > BMSCs*
7		8	F	ASCs > BMSCs	ASCs < BMSCs***	ASCs < BMSCs***	ASCs < BMSCs***	ASCs > BMSCs***
8		9	M	ASCs > BMSCs**	ASCs < BMSCs	ASCs < BMSCs*	ASCs < BMSCs**	ASCs > BMSCs***
9		14	F	ASCs > BMSCs*	ASCs < BMSCs*	ASCs < BMSCs***	ASCs < BMSCs***	ASCs > BMSCs**

*ALP* alkaline phosphatase, *ASCs* adipose-derived stem cells, *BMSCs* bone marrow-derived stem cells, *M* male, *F* female

*p < 0.05, **p < 0.01, ***p < 0.001

## Results

### ASCs and BMSCs shared a similar morphology and immunophenotype with small differences

**Fig. 1:**
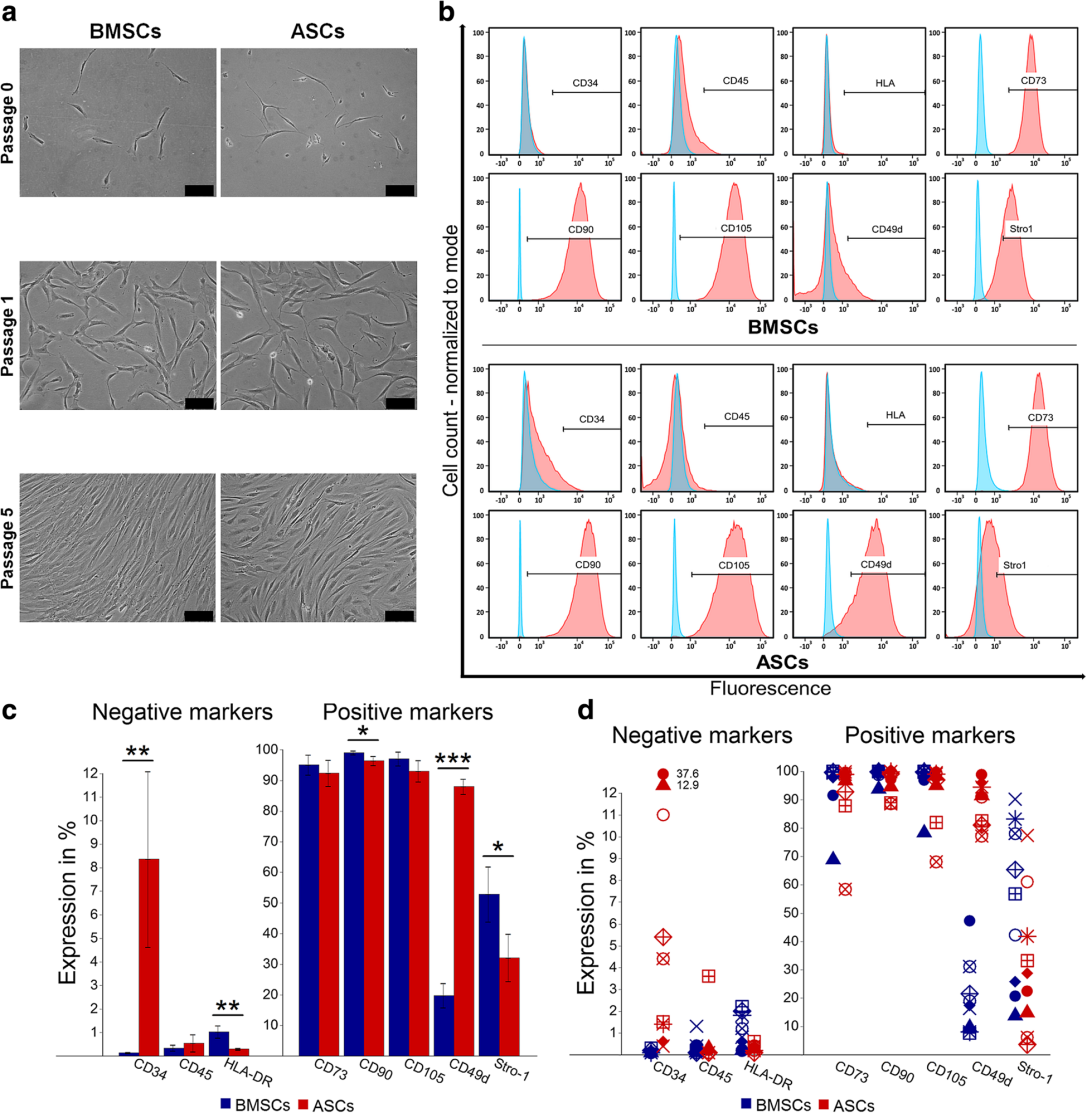
Morphology and immunophenotype characteristics of BMSCs and ASCs. **a** Representative microscopic illustrations of BMSCs and ASCs. Scale bar 100 μm. **b** Representative histograms from the flow cytometry analysis showing surface marker expression on BMSCs and ASCs, monoclonal antibody control (*blue*) and the stained cells (*red*). **c** and **d** Percentage of surface markers expression, average of nine donors (**c**) and in each donor (**d**). Each symbol represents one donor. *p < 0.05, **p < 0.01, ***p < 0.001. *ASCs* adipose-derived stem cells, *BMSCs* bone marrow-derived stem cells

ASCs and BMSCs were successfully isolated from the nine donors. Cells adhered to the plastic culture flask and were identified as ASCs and BMSCs ≈ 2 and ≈ 4 days after plating, respectively. ASCs and BMSCs showed a fibroblast-like cell morphology. After passage 0, ASCs reached 80% confluence after ≈ 6 days, compared to ≈ 8 days for BMSCs. During sub-culturing up to passage 5, no morphologic or growth pattern changes were observed (Fig. 1a). The stem cell markers CD73, CD90, and CD105 were generally highly expressed (> 90%) in ASCs and BMSCs, but the expression of these markers was less than 90% in ASCs in two of nine donors and in BMSCs in one of nine donors. ASCs and BMSCs had negative expression (≤ 2%) of the hematopoietic markers CD45 and HLA-DR, except for ASCs in one of nine donors. While BMSCs demonstrated negative expression (≤ 2%) of CD34, the average expression of this marker was significantly higher in ASCs (*p* < 0.01), and only ASCs in four of nine donors had expression ≤ 2%. The expression of CD49d in ASCs was significantly higher than in BMSCs (*p* < 0.001), which showed low expression (10–50%) in nine of nine donors. On average, there was a significant difference in the expression of Stro-1 in favor of BMSCs compared to ASCs (*p* < 0.05), but ASCs in one of nine donor demonstrated higher expression and the expression was almost similar in three of nine donors (Fig. 1b-d).

### ASCs continued to proliferate up to 21 days, but not BMSCs

**Fig. 2:**
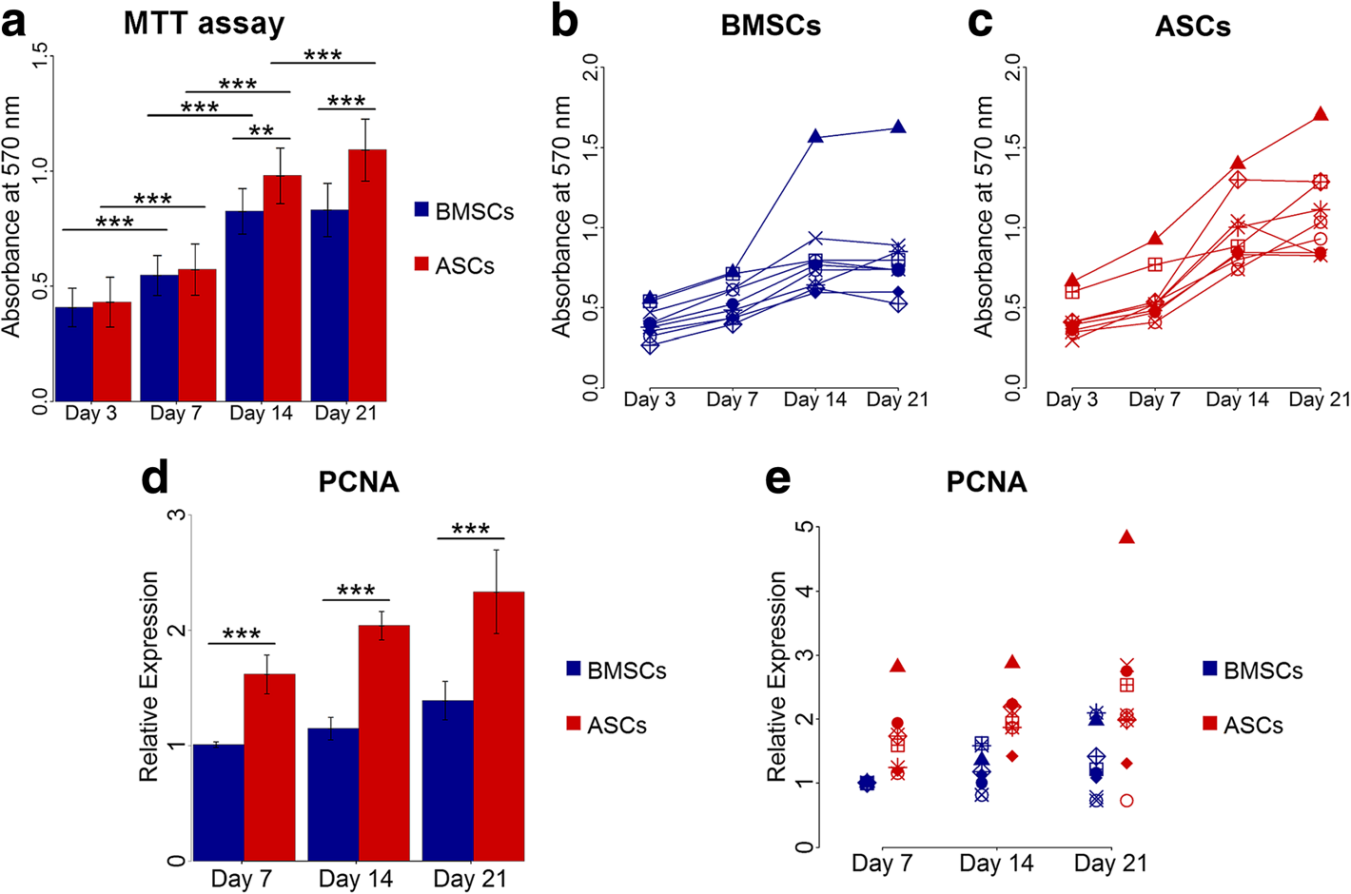
Proliferation of BMSCs and ASCs. **a-c** MTT assay at day 3, 7, 14 and 21, average of nine donors (**a**) and in each donor (**b** and **c**). **d** and **e** Relative gene expression of PCNA in BMSCs and ASCs at day 7, 14 and 21, average of nine donors (**d**) and in each donor (**e**). Each symbol represents one donor. **p < 0.01, ***p < 0.001. *ASCs* adipose-derived stem cells, *BMSCs* bone marrow-derived stem cells, *MTT* 3-(4,5-dimethylthiazol-2-yl)-2,5-diphenyl tetrazolium bromide, *PCNA* proliferating cell nuclear antigen

The MTT proliferation assay and expression of PCNA gene showed variability in the proliferation rate of ASCs and BMSCs among different donors (Fig. 2). Overall, the number of ASCs and BMSCs increased significantly with time from day 3 to 14 (*p* < 0.001). The proliferation rate of the stem cells from both sources was comparable at day 3 and 7. ASCs continued to proliferate significantly up to day 21 (*p* < 0.001), but proliferation reached a plateau from day 14 in BMSCs. Significantly higher cellular metabolic activity in ASCs compared to BMSCs was seen at day 14 and 21 (*p* < 0.01), indicating higher cell number (Fig. 2a-c). In addition, ASCs and BMSCs demonstrated upregulated expression of PCNA that increased with time from day 7 to 21. However, the expression was significantly higher in ASCs than in BMSCs (*p* < 0.001) (Fig. 2d and e). The higher metabolic activity in ASCs relative to BMSCs at day 21 was significant in five of nine donors (*p* < 0.05) (Table 2). The proliferation of ASCs and BMSCs was influenced by donor variations (Table 3).

**Table 3:** Effect of donor variation within ASCs and BMSCs using intra-class correlations (ICC) analysis

		ASCs ICC (95% Confidence interval)	BMSCs ICC (95% Confidence interval)
Proliferation		0.40 (0.17–0.69)	0.48 (0.23–0.74)
Osteogenic capacity	ALP activity	0.51 (0.23–0.78)	0.80 (0.58–0.92)
	Calcium deposition	0.98 (0.94–0.99)	0.98 (0.94–0.99)
Adipogenic capacity	Lipid vesicle formation	0.91 (0.79–0.97)	0.93 (0.83–0.97)

ICC value less than 0.40 low effect

ICC value from 0.40 to 0.74 moderate effect

ICC value from 0.75 to 1.00 high effect

*ALP* alkaline phosphatase, *ASCs* adipose-derived stem cells, *BMSCs* bone marrow-derived stem cells

### ASCs showed delayed ALP activity compared to BMSCs

**Fig. 3:**
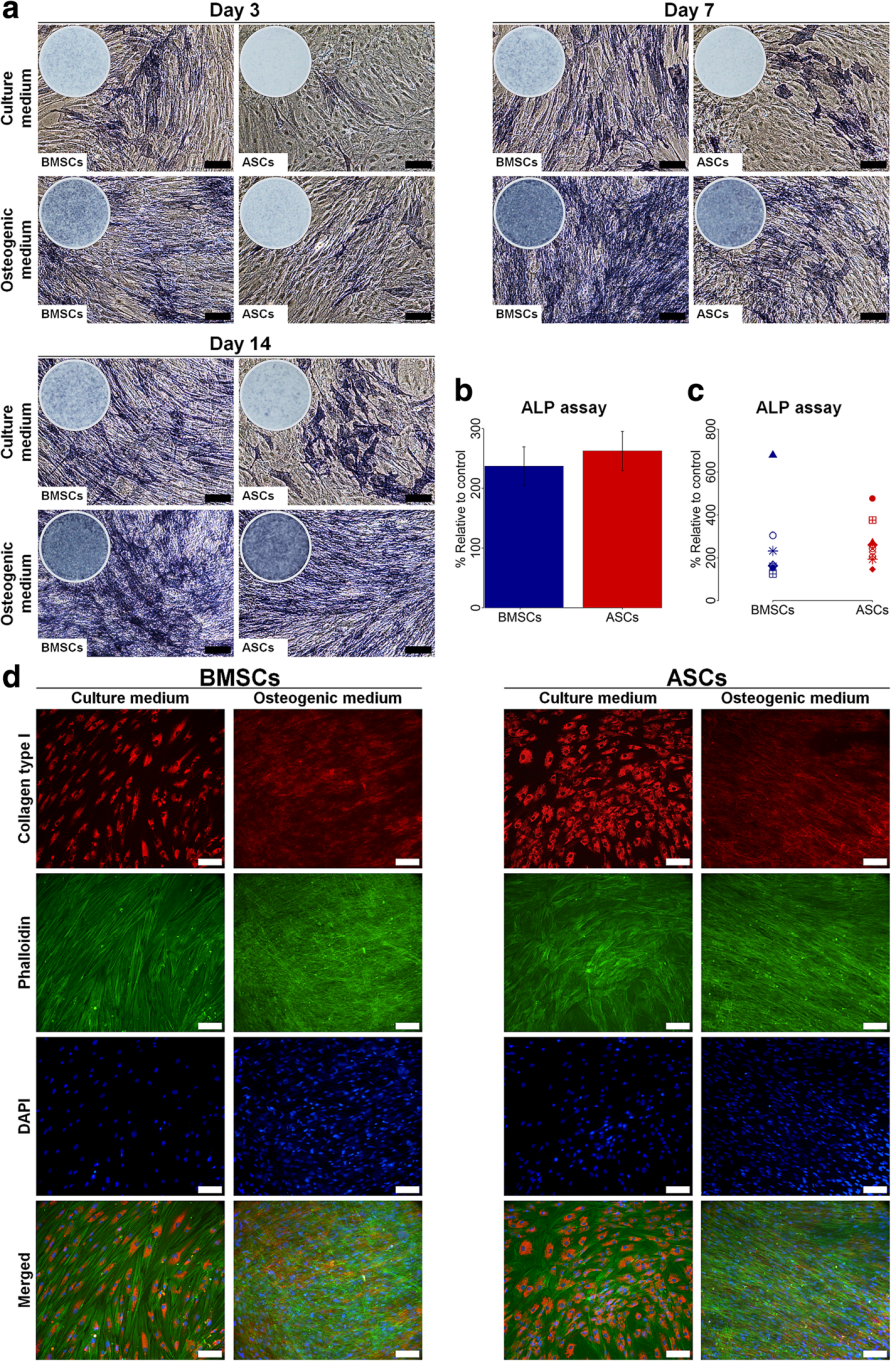
ALP activity and collagen type I formation in BMSCs and ASCs. **a** Representative images of ALP staining of BMSCs and ASCs at day 3, 7 and 14. Scale bar 100 μm. **b** and **c** ALP activity assay of BMSCs and ASCs at day 14, average of nine donors (**b**) and in each donor (**c**). **d** Representative images of IF staining of collagen type I in BMSCs and ASCs at day 14. Scale bar 100 μm. Each symbol represents one donor. *ALP* alkaline phosphatase, *ASCs* adipose-derived stem cells, *BMSCs* bone marrow-derived stem cells, *DAPI* 4′,6-diamidino-2-phenylindole

Overall, ALP activity in BMSCs cultured in osteogenic medium increased from day 3 to day 7, continuing to be high up to day 14 (Fig. 3a). For ASCs cultured in osteogenic medium, the ALP activity increased from day 3 to day 14, with a substantial increase in ALP activity observed at day 14, which was comparable to BMSCs. ALP activity in ASCs and BMSCs in control medium was less than for osteogenic medium, but with BMSCs showing more pronounced activity. The ALP assay showed variable activity in ASCs and BMSCs among different donors, from 1.3- to 7-fold for BMSCs and 1.5- to 5-fold for ASCs (Fig. 3b and c). Overall, no significant difference in ALP activity between ASCs and BMSCs at day 14 was detected. However, ALP activity was significantly higher in ASCs compared to BMSCs in three of nine donors (*p* < 0.05), whereas ASCs in two of nine donors had significantly less activity (*p* < 0.05) (Table 2). The ALP activity in ASCs and BMSCs was influenced by donor variations (Table 3).

### Comparable extracellular collagen type I was formed in ASCs and BMSCs

IF staining images showed that collagen type I was formed intracellularly as well as extracellularly by ASCs and BMSCs in osteogenic medium at day 14 from all donors (Fig. 3d). Only intracellular collagen type I was observed for ASCs and BMSCs in control medium. Overall, there was no remarkable difference in the formation of extracellular collagen type I between ASCs and BMSCs or between different donors.

### ASCs demonstrated delayed osteogenic capacity compared to BMSCs

**Fig. 4:**
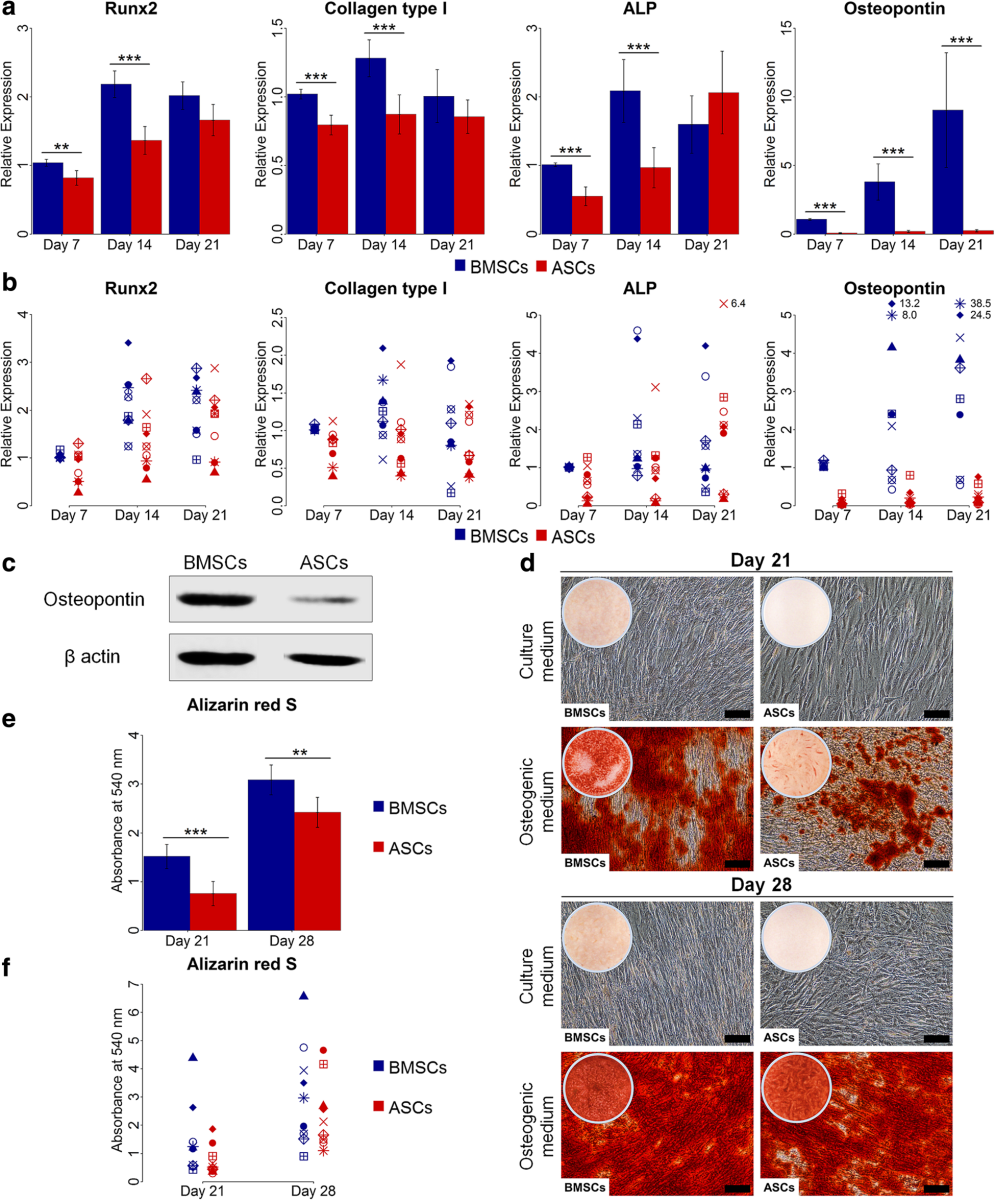
Osteogenic gene and protein expression, and calcium deposition in BMSCs and ASCs. **a** and **b** Relative gene expression of the osteogenic genes Runx2, collagen type I, ALP and osteopontin in BMSCs and ASCs at day 7, 14 and 21, average of nine donors (**a**) and in each donor (**b**). **c** Western blot analysis of osteopontin in BMSCs and ASCs at day 21. **d** Representative images after Alizarin red S staining of BMSCs and ASCs at day 21 and 28. Scale bar 100 μm. **e** and **f** Quantification of Alizarin red S staining of BMSCs and ASCs at day 21 and 28, average of nine donors (**e**) and in each donor (**f**). Each symbol represents one donor. **p < 0.01, ***p < 0.001. *ALP* alkaline phosphatase, *ASCs* adipose-derived stem cells, *BMSCs* bone marrow-derived stem cells, *Runx2 r*unt-related transcription factor 2

For the osteogenic differentiation at the gene level, evaluated by real-time qPCR, ASCs and BMSCs from the different donors showed variable expression of the osteogenic genes Runx2, collagen type I, ALP and osteopontin (Fig. 4a and b). Overall, the expression of Runx2, collagen type I and ALP increased from day 7 to day 14 in both ASCs and BMSCs, with significantly higher expression in BMSCs than in ASCs (*p* < 0.01). At day 21, no significant difference in the expression of these genes was detected. The expression of these genes in BMSCs was greatest at day 14. ASCs showed highest expression of Runx2 and ALP at day 21. The expression of osteopontin increased with time from day 7 to day 21, but was significantly higher in BMSCs than in ASCs (*p* < 0.001). ASCs showed low expression of osteopontin at all time points, in all donors. Similarly, western blot analysis at day 21 revealed higher osteopontin expression in BMSCs compared to ASCs (Fig. 4c). For the osteogenic differentiation evaluated by tissue-specific staining, ASCs and BMSCs showed osteogenic capacity, confirmed by Alizarin red S staining (Fig. 4d-f). Overall, BMSCs cultured in osteogenic medium at day 21 had significantly more calcium deposition compared to ASCs (*p* < 0.001). Although ASCs in osteogenic medium showed a remarkable increase in calcium deposition from 21 to 28 days, it was significantly lower than in BMSCs at day 28 (*p* < 0.01). A variation in calcium deposition in ASCs and BMSCs from different donors was detected. At day 21, ASCs showed significantly less calcium deposition than BMSCs in seven of nine donors (*p* < 0.05), and significantly more deposition in one of nine donor (*p* < 0.001) (Table 2). At day 28, two of nine donors had significantly higher calcium deposition in ASCs compared to BMSCs (*p* < 0.001), whereas five of nine donors had significantly less deposition in ASCs than in BMSCs (*p* < 0.01). ASCs and BMSCs cultured in control medium showed no calcium deposition. The calcium deposition in ASCs and BMSCs was highly influenced by donor (Table 3).

### ASCs showed chondrogenic capacity, but less than BMSCs

**Fig. 5:**
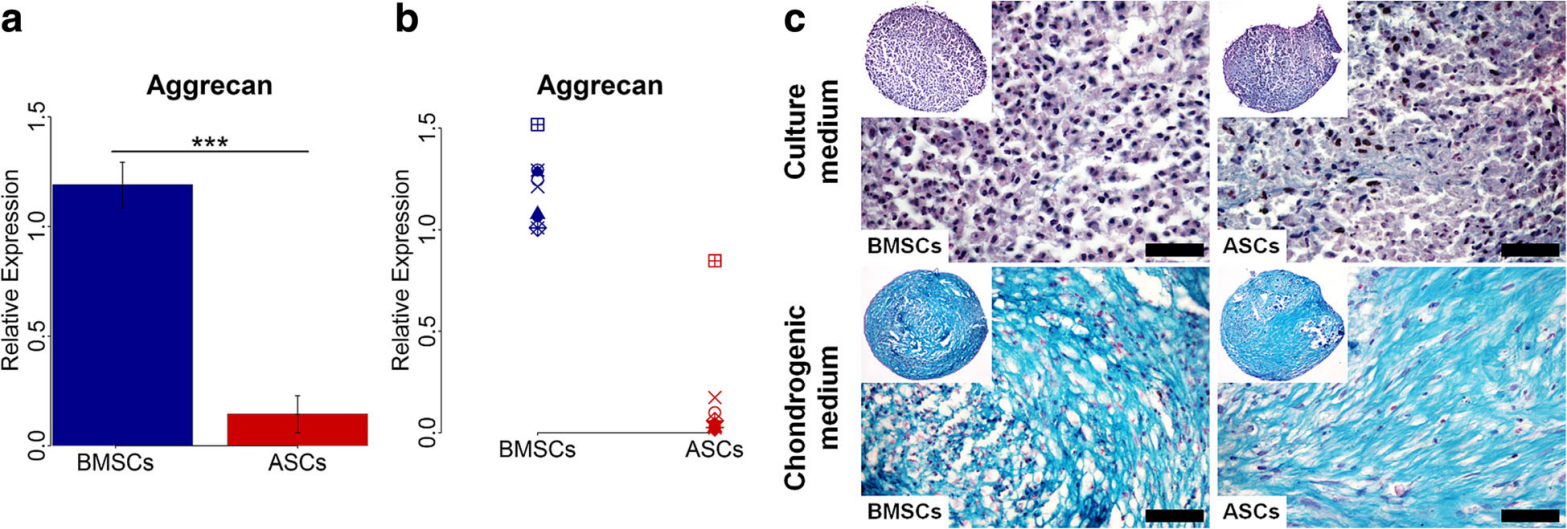
Chondrogenic gene expression and cartilaginous matrix formation in BMSCs and ASCs. **a** and **b** Relative gene expression of the chondrogenic gene aggrecan in BMSCs and ASCs at day 28, average of nine donors (**a**) and in each donor (**b**). **c** Representative images of Alcian blue-stained sections of BMSC and ASC pellets at day 28. Scale bar 50 μm. Each symbol represents one donor. ***p < 0.001. *ASCs* adipose-derived stem cells, *BMSCs* bone marrow-derived stem cells

Chondrogenic differentiation at the gene level, determined by real-time qPCR, showed that the expression of the chondrogenic gene aggrecan varied in ASCs and BMSCs among different donors (Fig. 5a and b). Overall, however, the expression was significantly higher in BMSCs than in ASCs (*p* < 0.001), with higher expression in BMSCs than ASCs in nine of nine donors. For chondrogenic differentiation, evaluated using tissue-specific staining, ASCs and BMSCs in chondrogenic medium formed pellets (diameter ≈ 1 mm) and showed chondrogenic capacity, confirmed by Alcian blue staining (Fig. 5c). After 28 days, the proteoglycan matrix of ASC and BMSC pellets were positively stained for Alcian blue. No remarkable difference in the cartilaginous proteoglycan matrix formation was noted between ASCs and BMSCs from all donors. No cartilaginous matrix was detected in ASC and BMSC pellets cultured in control medium.

### ASCs had greater adipogenic capacity than BMSCs

**Fig. 6:**
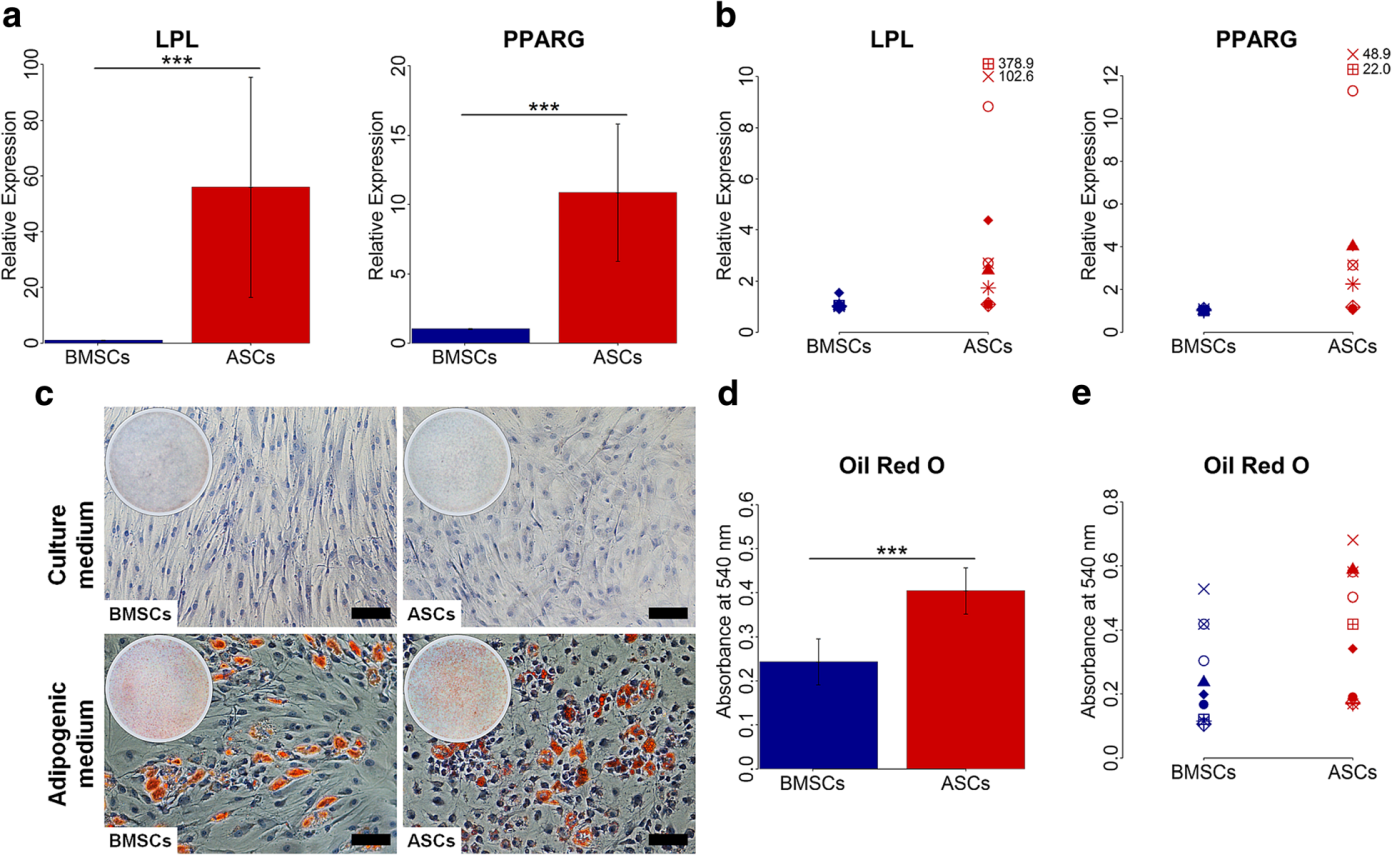
Adipogenic gene expression and lipid vesicle formation in BMSCs and ASCs. **a** and **b** Relative gene expression of the adipogenic genes LPL and PPARG in BMSCs and ASCs at day 14, average of nine donors (**a**) and in each donor (**b**). **c** Representative images of Oil red O stained BMSCs and ASCs at day 14. Scale bar 100 μm. **d** and **e** Quantification of Oil red O-stained BMSCs and ASCs at day 14, average of nine donors (**d**) and in each donor (**e**). Each symbol represents one donor. ***p < 0.001. *ASCs* adipose-derived stem cells, *BMSCs* bone marrow-derived stem cells, *LPL* lipoprotein lipase, *PPARG* peroxisome proliferator activated receptor gamma

Adipogenic differentiation at the gene level, evaluated by real-time qPCR, showed variation in the expression of the adipogenic genes in ASCs and BMSCs among different donors (Fig. 6a and b). Nevertheless, ASCs had significantly higher expression of the adipogenic genes LPL and PPARG compared to BMSCs at day 14 (*p* < 0.001), and this was observed in all donors. For adipogenic differentiation, evaluated using tissue-specific staining, ASCs and BMSCs in adipogenic medium showed adipogenic capacity, confirmed by Oil red O staining (Fig. 6c-e). When ASCs and BMSCs were cultured in adipogenic medium, the cells started to lose their spindle-like morphology and lipid vesicles started to form in the cytoplasm. After 14 days, both ASCs and BMSCs showed positive Oil red O stained intracellular lipid vesicles. ASCs cultured in adipogenic medium demonstrated significantly higher amounts of intracellular lipid vesicles than BMSCs (*p* < 0.001), a finding observed in nine of nine donors (*p* < 0.05) (Table 2). No formation of lipid vesicles was detected in ASCs and BMSCs cultured in control medium. The lipid vesicle formation in ASCs and BMSCs was highly influenced by donor (Table 3).

## Discussion

Adult MSCs, isolated from different connective tissues, have a fundamental role in maintenance and regeneration of body tissues [30]. The density, proliferation and differentiation capacity of MSCs derived from different sources are crucial in cell-based therapy. In this study, we compared the surface marker expression, proliferation and differentiation capacity of donor-matched ASCs and BMSCs derived from young patients. This reduces the possibility of biological variations experienced when comparing cells from different individuals, raising questions about the reliability of the comparison [22]. ASCs and BMSCs were derived from a homogenous age group to avoid heterogeneity in age and to limit possible age-related differences [28]. It has been reported that stem cell yield, proliferation and differentiation properties of BMSCs and ASCs are negatively affected by age [31, 32]. However, conflicting conclusions on the effects of aging on multi-lineage differentiation capacity of MSCs have been reported [33–35]. Different isolation methods of BMSCs have been described, but the direct plating method is simple and advantageous compared to other methods [29, 36]. Isolation of ASCs from an adipose tissue block is reported superior to liposuction [8, 11]. Immediately after isolation, we noted heterogeneous cell populations, with an increase in homogeneity during subsequent passages. Isolated ASCs and BMSCs showed the spindle fibroblast-like cell morphology and the capacity to adhere to culture flasks without notable morphologic changes during expansion up to passage 5. Flow cytometry showed a relatively similar immunophenotype of BMSCs and ASCs. The stem cell markers CD73, CD90 and CD105 were highly expressed and both cell types demonstrated low expression of the hematopoietic markers CD34, CD45 and HLA-DR. However, the expression of the surface marker CD34 was higher in ASCs than in BMSCs. This is in agreement with previous data, showing expression of CD34 in freshly isolated ASCs that gradually reduced during passaging, but was not totally lost [37]. The morphology and immunophenotype characteristics of the isolated BMSCs and ASCs were consistent with criteria proposed by the ISCT [4]. Previous findings have demonstrated strong expression of CD49d on ASCs [38–40], in line with our results showing a higher expression of CD49d in ASCs compared to BMSCs in all donors. The expression of Stro-1, a marker for cells multi-lineage potential [41], has been demonstrated in both BMSCs and ASCs [38, 39]. This was confirmed by the present study, but BMSCs had increased expression of Stro-1 compared to ASCs. Previous studies have reported a higher proliferation rate of BMSCs than for ASCs [12, 13]. The present study showed that ASCs continued to increase in number up to 21 days. The continued proliferation of ASCs may be linked to the expression of CD34 by ASCs, as CD34 is suggested to play a role in the long-term proliferation of MSCs [42]. This is supported by other studies, reporting similar or greater rates of proliferation of ASCs compared to BMSCs [18, 19, 21, 22, 24, 25, 43]. The decrease in the metabolic activity from day 14 to 21 in ASCs and BMSCs from some donors might be explained by contact inhibition of proliferation, which is the tendency of cells to stop proliferation when reaching confluence [44]. This might be associated with changes in cell size and cytoskeleton, eventually resulting in cell differentiation or apoptosis [45].

Differentiation toward different cell lineages confirms the multi-potency of the stem cells and hence their efficient therapy potentials. It has been reported that MSCs can be expanded up to passage 4 without losing their multi-potency [34]. This study showed multi-potency of both ASCs and BMSCs up to passage 5. The osteogenic differentiation of MSCs is characterized by proliferation, matrix maturation and mineralization [46]. Collagen type I is the most abundant protein in bone extracellular matrix (ECM). Formation of mature collagenous ECM is necessary for mineralization through the deposition of minerals like calcium and phosphate [46]. In the presence of a collagen matrix, ALP enzyme activity is essential for initiation of mineralization [47]. The formation of extracellular collagen type I, in addition to earlier high ALP activity in BMSCs, compared to ASCs, indicate earlier maturation of BMSCs than ASCs during osteogenic differentiation. ASCs might have the highest ALP activity later than day 14. In order to compare the in vitro osteogenic differentiation of ASCs and BMSCs at the gene level, we compared the gene expression of four osteogenic lineage-specific genes. Runx2 is an early osteogenic marker essential for osteoblast differentiation and regulates other osteogenic genes, e.g. collagen type I, ALP and osteopontin [48, 49]. Runx2-deficient mice are reported to lack bone formation due to absence of osteoblasts [50]. Runx2 and collagen type I genes are expressed during the proliferation stage of osteoblast differentiation, but the expression declines and remains at low level during maturation and mineralization [46, 49]. The ALP gene level increases and reaches a peak during matrix maturation and decrease in proliferation activity, followed by a subsequent decline during mineralization [46]. Osteopontin is an important non-collagenous organic component of bone matrix. Low level expression of this gene can be detected in the early stage of osteogenic differentiation, and the expression increases and peaks with mineralization [46, 49]. Our results revealed that BMSCs expressed the early osteogenic genes Runx2, collagen type I and ALP, and the expression was highest at day 14. In contrast, the expression of Runx2 and ALP was highest at day 21 in ASCs. This suggests that BMSCs stopped proliferation and started differentiation and formation of a mature collagenous matrix at day 14. By comparison, ASCs showed an extended proliferation stage and delayed differentiation and mature matrix formation. This is supported by the results from the proliferation assay, showing that ASCs continued to proliferate until day 21. The finding that BMSCs stopped proliferation at day 14 supports a conclusion of earlier osteogenic differentiation in BMSCs than in ASCs. This conclusion was further strengthened by upregulated expression of osteopontin in BMSCs at day 21 compared to low expression in ASCs at the gene level, and confirmed at the protein level for the same time point. Moreover, results of Alizarin red S staining at day 21 showed significantly more calcium deposition for BMSCs than ASCs, indicating earlier mineralization of the ECM. Abundant mineralization was observed at day 28 for ASCs. ASCs and BMSCs with higher ALP activity demonstrated more calcium deposition than counterparts from donors with lower ALP activity. This is in agreement with the fact that matrix mineralization is strongly correlated with ALP enzyme activity [27, 34]. This is also in agreement with a recent report that compared donor-matched ASCs and BMSCs, showing that BMSCs exhibited higher in vitro osteogenic capacity than ASCs based on calcium deposition and expression of osteogenesis-related genes [23]. However, in that study ASCs and BMSCs were obtained from donors older than 60 years. Other studies have also shown that BMSCs have superior in vitro osteogenic capacity than ASCs with respect to higher ALP activity, calcium content and expression of the early and late osteogenic genes [21, 22, 43]. However, others have reported that the in vitro osteogenic differentiation of ASCs is superior or similar to BMSCs in terms of calcium deposition and expression of osteogenesis-related genes [18–20, 25, 51]. Moreover, the osteogenic capacity of ASCs was enhanced under dynamic culture conditions subjected to mechanical stimulation [52] and under culture conditions supplemented with platelet-derived growth factor [53, 54], vitamin D3 and bone morphogenetic protein-2 (BMP-2) [55]. Aggrecan is a major component of cartilage extracellular matrix [56]. ASCs and BMSCs differentiated to a chondrogenic lineage. However, BMSCs showed significantly higher expression of the cartilage-specific gene aggrecan than ASCs at day 28, similar to results that have previously been published [12, 57]. LPL and PPARG are highly expressed in adipose tissue, and PPARG is essential for the adipocyte differentiation [58, 59]. With regard to adipogenic differentiation, ASCs demonstrated significantly higher lipid vesicle formation and expression of the adipogenic lineage-related genes LPL and PPARG compared to BMSCs, which is in line with previous findings [60]. These findings indicate that ASCs and BMSCs have tissue-specific differentiation potential, since higher in vitro osteogenic and chondrogenic potential was observed in BMSCs, and higher adipogenic capacity was found in ASCs. Previous reports have also concluded that MSCs preferentially differentiate into cells of the same tissue origin [12, 60].

Our results also confirmed that the proliferation and differentiation capacity of both ASCs and BMSCs varied by donor. The harvested population from each bone marrow and adipose tissue sample is not uniform and may thus contain MSCs and cells at different differentiation stages, with different proportions and with variations from donor to donor [34], not only in donors with variations in age as previously reported [34], but also in donors with similar age as shown in this study. A pure population of MSCs will possibly have better proliferation and multi-lineage differentiation capacity than a heterogeneous population consisting of MSCs and cells at different differentiation stages. The tissue sampling method might also affect the heterogeneity of the MSCs population and be responsible for donor-to-donor variations, as MSCs properties varies between samples obtained from the same donors at different times [27]. Unidentified factors in the medical history and the physiological status of the donors might also contribute to variations in the in vitro properties of ASCs and BMSCs [27, 34]. Furthermore, epigenetic regulation factors are reported to influence the osteogenic and adipogenic differentiation capacity of BMSCs and ASCs. This is based on the DNA methylation status of the main transcription factors Runx2 and PPARG that control the fate of MSCs [23].

## Conclusions

In this study, we compared the in vitro properties of ASCs and BMSCs derived from the same nine individuals. Overall, ASCs and BMSCs were comparable with regard to morphology and immunophenotype. ASCs and BMSCs demonstrated multi-potency; nevertheless, their differentiation capacity varied, with tissue-specific differentiation seen. BMSCs were superior to ASCs in terms of osteogenic and chondrogenic differentiation, while ASCs had higher proliferation and adipogenic potential. We also found that the donor is an important factor influencing the properties of MSCs, since properties of MSCs derived from adipose tissue and bone marrow varied by donor. These similarities and differences in ASCs and BMSCs should be taken into consideration when planning stem cell-based clinical therapy.

## Abbreviations

ALP: Alkaline phosphatase
ASCs: Adipose-derived stem cells
BMP-2: Bone morphogenetic protein-2
BMSCs: Bone marrow mesenchymal stem cells
BSA: Bovine serum albumin
DAPI: 4′,6-diamidino-2-phenylindole
DMEM: Dulbecco’s modified Eagle’s medium
DMSO: Dimethyl sulfoxide
ECM: Extracellular matrix
FBS: Fetal bovine serum
GAPDH: Glyceraldehyde-3-phosphate dehydrogenase
ICC: Intra-class correlation
IF: Immunofluorescence
LPL: Lipoprotein lipase
MSCs: Mesenchymal stem cells
PBS: Phosphate-buffered saline
PCNA: Proliferating cell nuclear antigen
PPARG: Peroxisome proliferator activated receptor gamma
PVDF: Polyvinylidene difluoride
qPCR: Quantitative polymerase chain reaction
Runx2: Runt-related transcription factor 2
SDS: Sodium dodecyl sulfate
